# Fuzzy Mixed Assembly Line Sequencing and Scheduling Optimization Model Using Multiobjective Dynamic Fuzzy GA

**DOI:** 10.1155/2014/505207

**Published:** 2014-03-27

**Authors:** Farzad Tahriri, Siti Zawiah Md Dawal, Zahari Taha

**Affiliations:** ^1^Centre for Product Design and Manufacturing, Department of Mechanical Engineering, Faculty of Engineering, University of Malaya, 50603 Kuala Lumpur, Malaysia; ^2^Faculty of Mechanical Engineering, Universiti Malaysia Pahang, 26600 Pekan, Pahang Darul Makmur, Malaysia

## Abstract

A new multiobjective dynamic fuzzy genetic algorithm is applied to solve a fuzzy mixed-model assembly line sequencing problem in which the primary goals are to minimize the total make-span and minimize the setup number simultaneously. Trapezoidal fuzzy numbers are implemented for variables such as operation and travelling time in order to generate results with higher accuracy and representative of real-case data. An improved genetic algorithm called fuzzy adaptive genetic algorithm (FAGA) is proposed in order to solve this optimization model. In establishing the FAGA, five dynamic fuzzy parameter controllers are devised in which fuzzy expert experience controller (FEEC) is integrated with automatic learning dynamic fuzzy controller (ALDFC) technique. The enhanced algorithm dynamically adjusts the population size, number of generations, tournament candidate, crossover rate, and mutation rate compared with using fixed control parameters. The main idea is to improve the performance and effectiveness of existing GAs by dynamic adjustment and control of the five parameters. Verification and validation of the dynamic fuzzy GA are carried out by developing test-beds and testing using a multiobjective fuzzy mixed production assembly line sequencing optimization problem. The simulation results highlight that the performance and efficacy of the proposed novel optimization algorithm are more efficient than the performance of the standard genetic algorithm in mixed assembly line sequencing model.

## 1. Introduction

Mixed-model assembly lines (MMAL) have been widely used by manufacturers and they play a key role in the production of a variety of products. Products with similar characteristics are assembled with different processing times on the same assembly line at very low costs [1–3]. MMAL reduce setup operations to an extent that various models from a common base product can be manufactured in intermixed sequences. Mixed-model sequencing (MMS) aims at avoiding or minimizing sequence-dependent work overload based on detailed scheduling which explicitly accounts for operation time, worker movement, station borders, and other operational characteristics of the line [4]. MMS is an NP-hard problem which requires a unique and stable model to facilitate the production of mixed products in a manufacturing environment with multiple parts, machines, products, and assemblies in order to minimize scheduling time, idle time of the machines, setup number, and setup cost as well as maximize the number of products and assembly of various products [2, 3].

A contribution of this paper is to develop a new multiobjective fuzzy genetic algorithm model by integrating fuzzy expert experience controller (FEEC) with automatic learning dynamic fuzzy controller (ALDFC), which combines the execution time of GAs with dynamic control of population size, number of generations, tournament candidate, crossover rate, and mutation rate in order to solve a fuzzy mixed-model production line sequencing problem. The line-sequencing optimization model is based on two objectives such as minimizing a make-span and minimizing the machine setup number of the production line. The significance of this model is for those factories who want to produce various kinds of products with fixed machine just by changing the sequencing of the products. The model helps the manager to sequence and schedule the production line easily and accurately by taking the market demand into consideration.

## 2. Related Works

A number of studies attempted at solving multi- and mixed-product assembly lines sequencing problems using sequencing mathematical procedures and simulation models that will optimize various system measures such as throughput, scheduling time, number of stations, idle time, flow time, line length, work-in-process, and raw material demand deviations [1, 5–17].

In the advent of metaheuristic algorithms in recent years, numerous complex scheduling problems have been studied and solved using metaheuristic search techniques such as ant colony optimization (ACO), tabu search (TS), genetic algorithm (GA), and simulated annealing (SA). Metaheuristic algorithms are used to overcome the complexity of sequencing in assembly line problems [18].

Early research on the use of genetic algorithm (GA) for mixed-model line sequencing problems was carried out by Ghosh and Gagnon [19], whereby they introduced a mathematical programming model and an iterative GA-based procedure for MALBP with parallel work station. The goal of their research was to maximize the production rate of the line for a predetermined number of operators. A spreading and cutting sequencing (SCS) model using GA was implemented by Wong et al. [20] to solve the sequencing problem by reducing the completion time for daily operation of fabric spreading and cutting as well as improving the utilization of a computerized fabric cutting system used in the garment industry. Moon et al. [21] proposed an integrated machine tool selection and sequencing model to optimize the total production time and workload between machine tools using GA. In recent research, Norozi et al. [18] developed an intelligence-based GA in order to tackle the complexity of sequencing in parallel MMAL.

Most of the real-world decision problems involve multiple conflicting objectives that need to be tackled while adhering to the various constraints [22, 23]. Multiobjective optimization in mixed-model assembly line sequencing is dependent on the setup between product variants such as setup number, time, and cost as well as on the best sequence of total utility work to minimize the completion scheduling time (make-span) simultaneously. A number of studies used multiobjective optimization in mixed-model assembly line sequencing as follows: [2, 3, 24–30].

Genetic algorithm (GA) has been proven to be highly effective for achieving optimum or near-optimum solutions to complex real-world multiobjective optimization problems. However, GAs are limited by the fact that their performance is very sensitive to parameter settings. GA design consists of two key steps, namely, genetic operations and parameter settings [31]. The genetic operations involve choosing a suitable selection method. Parameter settings involve setting the required parameters and variables for controlling the algorithms such as population size, number of generations, number of selected candidates, crossover rate, and mutation rate [32]. Adaptive genetic operators (AGOs) are used in GA design for controlling a parameter. AGO can be classified into two modes, whereby the first mode involves implementing artificial intelligence techniques such as FLCs, and the second mode involves implementing conventional heuristics. The GA parameters controlled by these two modes are regulated adaptively during the genetic search process. This yields significant time savings during fine-tuning of the parameters, and the capabilities of GAs can be improved in searching for a global optimum [23, 33]. In adaptive genetic operators (AGOs) using fuzzy logic control, the fuzzy rules describing the relationship between the inputs and outputs need to be defined once the rule base has been determined. The rule base is determined after the inputs and outputs are selected and the database has been defined. There are various ways to achieve this objective and one way is to use the knowledge and experience of GA experts or by implementing an automatic learning technique for cases where knowledge and expertise are unavailable [34, 35]. Several works were focused on AGOs using artificial intelligent techniques for adaptive selection, crossover, and mutation in which the rules are based on the knowledge and experience of GA experts, as follows: [31, 32, 36]. A number of works on AGOs using artificial intelligent automatic learning technique rules include those of [35, 37–40]. Wang et al. [41] developed a fuzzy logic controlled genetic algorithm (FCGA) for environmental/economic dispatch. They proposed an improved genetic algorithm with two FLCs (one for crossover rate and mutation rate, resp.) based on several heuristics during the optimization process. The main concepts were implemented independently to adaptively regulate the crossover and mutation rates during the genetic search process. These parameters were taken as the input variables of the GAs, as well as the output variables of the FLCs.

It can be observed from the literature review that a few papers addressed mixed-assembly line sequencing and therefore there is a need for a detailed investigation on mixed-assembly line sequencing. It is found that the production of mixed-models is influenced by a number of criteria, which limit the applicability of the research results in real production line conditions. It shall be highlighted that only one criterion was considered in previous works such as operation line, and therefore other factors such as the travelling time of the conveyor were neglected. Deterministic timing has also been of interest in most studies. It shall be highlighted that only assembly line balancing was carried out in previous works in order to minimize the prediction time for input data, which include the travelling and processing times for each available job. Although the results from mixed-assembly line sequencing studies are applicable in real manufacturing environments, little is known on fuzzy mixed-model assembly line sequencing. Hence, it is evident that there is a lack of studies which implements the concept of fuzzy time in order to minimize the predicted time of input data. In general, the research papers can be classified into two groups, whereby the first group focuses solely on objective criteria, while the second group focuses on multiobjective investigations. A critical evaluation of previous works clarifies that addressing these objectives involves the development of various methods, in which MMAL sequencing is the ideal method for a single objective. Comparison of various GA methods reveals that multiobjective studies have not been investigated extensively, unlike single-objective studies. Much effort has been made to intensify and accelerate the running of GA methods to achieve optimum results. Although mixed-model assembly line sequencing is of prime importance, there is a lack of studies which focus on this topic. In this paper, a hybrid method is proposed, in which mixed-model assembly line sequencing is integrated with the operating and travelling time in the form of fuzzy numbers of a multiobjective optimization problem. It can be observed from the existing literature that much effort has been devoted to intensify and accelerate the execution of GAs to attain optimum results. A number of works were focused on FGAs, in particular, the augmentation of GAs using fuzzy logic. Several works were focused on improving the performance of FGA methods. It shall be highlighted that these works primarily consider proliferating certain control parameters in GAs such as the identification of the population size, number of generations, tournament candidate, crossover rate, and mutation rate. Accelerating the speed of these parameters is carried out dynamically. The authors noted that there exists a gap in these works, whereby the mixed model assembly line sequencing is overlooked for each GA run. In light of the above review, this study is aimed at developing a new multiobjective fuzzy genetic algorithm by integrating fuzzy expert experience controller (FEEC) with automatic learning dynamic fuzzy controller (ALDFC), which combine the execution time of GAs with dynamic control of population size, number of generations, tournament candidate, crossover rate, and mutation rate in order to solve a fuzzy mixed-model assembly line sequencing problem.

## 3. Multiobjective Sequencing Problem in Mixed Model Assembly Line

**Figure 1:**
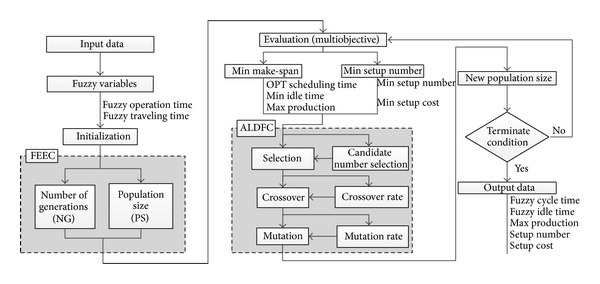
Flowchart of the proposed multiobjective fuzzy mixed assembly line sequencing model by using fuzzy genetic algorithm approach.

The procedure of the proposed multiobjective fuzzy mixed assembly line sequencing model by integrating a fuzzy expert experience controller (FEEC) with automatic learning dynamic fuzzy controller (ALDFC) technique in genetic algorithm is shown in Figure 1. These steps are carried out for various types of applications and include input data, fuzzy variables, initialization, evaluation, selection, crossover, mutation, and termination. The input data are coded by considering the mixed model assembly line sequencing during the initialization of parameters. The operation time and travelling time are represented by fuzzy numbers. The fuzzy expert experience controller is used in order to determine the number of generations and population size in genetic algorithms. The fitness function values are grouped into two main objectives, that is, minimizing the make-span and setup number. The automatic learning dynamic fuzzy controller technique was adopted in order to dynamically control the genetic operators based on existing conditions such as selecting the number of tournament candidates as well as setting the crossover and mutation rates. Nine rules are used to control the crossover rate during the crossover stage, whereas eight rules are used to control the mutation rate during the mutation stage. Two rules are designed for the termination stage in order to control termination and gain optimum results. 

The model is developed based on the works of [2, 24, 28, 42–44]. Moreover, the main references which support the development of the method in this research are [31, 33, 37, 45, 46].

### 3.1. Mixed Model Assembly Line Sequencing (MMALS)

The input data involves identifying the number of machines (*M*_*i*_) for producing the parts (*P*_*i*_) or products (P.NO_*i*_) such as CNC, NC, and Robot as well as assigning the parts to their respective machines and robots based on the production and assembly line sequence (A.S_*i*_).

The processing time $\left(\tilde{\text{OP}}\right)$ of each robot and machine as well as the travelling time $\left(\tilde{\text{T}.\text{t}}\right)$ of each part assigned to the machines is represented as fuzzy numbers in the mixed-model assembly line sequencing model, rather than deterministic time values. The output data will become more accurate and representative of the real-case data by implementing trapezoidal fuzzy numbers (TFNs). The same procedure used by Fonseca et al. [47] is adopted in order to adapt the deterministic operation and travelling time in the fuzzy domain.

Initialization of parameters involves setting the parameters of the GA, creating the scores for the simulation, and creating the first generation of chromosomes. A total of 18 parameters are set during initialization, as shown in Table 1.

**Figure 2:**
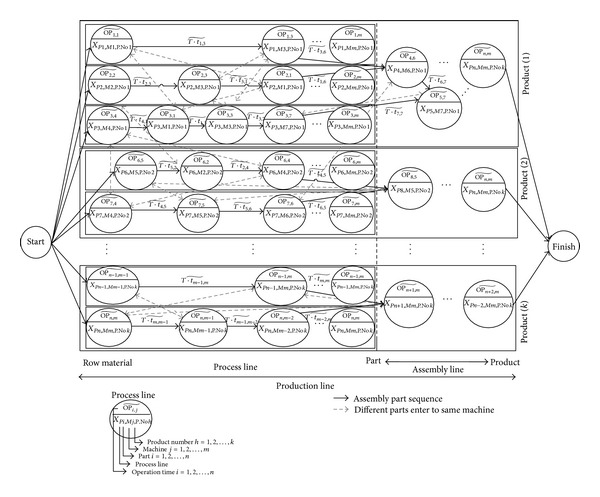
Example of a general mixed-model assembly line problem.

A general model for the mixed-model assembly line sequencing problem is shown in Figure 2, in which the parameters are listed in Table 1. The main solid arrows (→) in Figure 2 represent the sequence to produce the parts, followed by a discrete part manufacturing assembly, leading to the final product. Likewise, the dashed lines (- - ->) represent the inputs to the same machine. The elapsed time between machines in order to manufacture a part is given by $\left(\tilde{\text{T}.\text{t}}j,j+1\right)$. The results of the model are dependent on the difficulties encountered during production planning and sequence. The number of genes in the chromosome (*X*_*i*_) is formed based on the job numbers (*J*_*i*_). Once the chromosomes have been formed, the chromosomes will be filled with random numbers via stochastic repeating, neglecting the encoding sequence of the produced chromosomes. The numbers vary between 0 and the maximum number of genes in the chromosomes (*X*_*n*_). Once the chromosomes have been filled with random numbers, the classified gene code numbers are ranked into a possible sequence which can be used by the GA. The steps are described briefly as follows. 

#### 3.1.1. Identification of the First of Each Part Type's Gene Code

The first of each part type's gene code is identified using (1), whereby each part has a sequence of gene codes with *k*-elements (*M*_1_, *M*_2_,…, *M*_*k*_). The values for *M*_1_, *M*_2_,…, *M*_*k*_ are given:

(1) $$\begin{array}{c} X_{i}-M_{1}\leq \left(k-1\right). \end{array}$$

#### 3.1.2. Classification Based on the Product's Part Sequence

Each gene code number in the chromosome line is compared to the previous one. Three conditions are used when comparing the gene code numbers, as described below.

- The first or existing gene code number fills up the new chromosome without any changes in the gene code number.
- If the gene code number is the same as another gene code number within the chromosome, the chromosome is filled up with the addition of one number out of the existing gene code numbers.
- If the gene code number is different from other gene code numbers within the chromosome, the existing gene code number fills up the chromosome without any changes in the gene code number.

#### 3.1.3. Classification Based on the Assembly Sequence

Once the product's part sequence has been classified, the chromosome is filled up using the assembly sequence number related to a gene code (*X*_*i*_) of the specific chromosome. Finally, the final gene code numbers are ranked by two sequence filters, which accounts for the assembly sequence code and product's part sequence. 

### 3.2. Objective Functions

The fitness function values for multiobjective mixed-production assembly line sequencing are categorized into two main objectives, that is, minimizing the total make-span and setup number. Each chromosome is evaluated during each generation of the selection process. This is accomplished by looking up the score of each gene in the chromosome, adding the scores, and averaging the scores for the chromosome. The elite chromosome of the generation is determined as part of the evaluation process. The following assumptions are made for the multiobjective evaluation.

1. The conveyor (operator movement) moves at a constant speed. If job overlapping occurs, the remaining work will be accomplished by temporary operators.
2. The job operation begins when the part enters the machine. Once the job is completed, the operator will move the part to the next machine.
3. The operator is assigned to each selected part that is assigned to each machine.
4. The position of the machines on the assembly line varies from one to another depending on the user input, which is based on the travelling time.
5. The assembly line can process jobs for a product family, which is described by a joint priority matrix.
6. The processing time varies for different jobs and these jobs are allocated to the same machine. However, what is the optimum processing time for each job?
7. The demand for all products and the sequence of the products entering the assembly line are predetermined.
8. The completion time for all jobs is represented by a fuzzy number.

#### 3.2.1. Minimizing the Total Make Span

The step involves evaluating each chromosome based on the make-span by considering the product number and assembly sequence for a gene code (*X*_*i*_). The *x*_1_, *x*_2_, *x*_3_, and S.t(*X*_*i*_) are computed in which *X*_*i*_ is calculated as follows. *x*_1_ is determined by checking the start time of the parts entering the machines, based on the sequence assigned to the machines. This is expressed by

(2) x1={0,P(Xi)≠P(Xk),S.t(Xk)+OP(Xk)+T.t(Xk),P(Xi)=P(Xk),k=i−1,  i−2,…,2,1,

where  *X*_2_ is determined by checking the start time of the parts entering the machines based on the assigned machines, as given by

(3) x2={0,M(Xi)≠M(Xk),S.t(Xk)+OP(Xk),M(Xi)=M(Xk),k=i−1,i−2,…,2,1,

where  *x*_3_ is then determined by checking the start time of the parts entering the machines based on the sequence of the part's assembly for each product, as given by (6) and (7). 

For each *X*_*i*_ where *i* = 1,2,…, *n*, note that the product number (P.No(*X*_*i*_)) and assembly sequence (A.S(*X*_*i*_)) are important. Equations (4) through (7) are given below.

(i) (4) $$\begin{array}{c} \text{If}\;\;\text{A}.\text{S}\left(X_{i}\right)=0⟹x_{3}=0,\;i=1,2,\ldots ,n, \end{array}$$
  (5) $$\begin{array}{c} \text{If}\;\;\text{A}.\text{S}\left(X_{i}\right)\neq 0⟹\text{compares}\;\;\text{P}.\text{No}\left(X_{i}\right),\;\;\text{P}.\text{No}\left(X_{i-1}\right). \end{array}$$
(ii) If P.No(*X*_*i*_) = P.No(*X*_*i*−1_), then
  (6) x3={S.t(Xi−1), A.S(Xi)=A.S(Xi−1),S.t(Xi−1)+OP(Xi−1)+T.t(Xi−1), A.S(Xi)≠A.S(Xi−1),i=1,2,…,n.
(iii) If P.No(*X*_*i*_) ≠ P.No(*X*_*i*−1_) when searching a gene code *X*_*k*_, where *k* = 1,2,…, *i* − 2, go backwards one by one until P.No(*X*_*i*_) = P.No(*X*_*k*_) is met:
  (7) x3={S.t(Xk), A.S(Xi)=A.S(Xk),S.t(Xk)+OP(Xk)+T.t(Xk), A.S(Xi)≠A.S(Xk),i=1,2,…,n.

The start time is obtained using (8), in which *x*_1_, *x*_2_, *x*_3_ are determined from the previous steps:

(8) $$\begin{array}{c} \text{S}.\text{t}\left(X_{i}\right)=\mathrm{Max}\left(x_{1},x_{2},x_{3}\right);\;X_{i},\;\;i=1,2,\ldots ,n. \end{array}$$

*Note*. In the first run, if *x*_1_ = *x*_2_ = *x*_3_ = 0, then S.t = *Max*⁡(*x*_1_, *x*_2_, *x*_3_) + T.t.

The make-span fitness function is then calculated for all chromosomes using

(9) Final  scheduling  time=Max⁡ S.t(Xi)+OP(Xi)              i=1,2,…,n.

#### 3.2.2. Minimizing the Setup Number

The machine number *M*(*X*_*i*_) and setup number S.No(*X*_*i*_) are defined for element (*X*_*i*_) in the chromosome, where *i* = 1,2,…, *n*, once *M*(*X*_*i*_) and *S*.No(*X*_*i*_) have been determined for all *X*_*i*_ (where *i* = 1,2,…, *n*). If *M*(*X*_*i*_) = *M*(*X*_*j*_) = *t* (where *i*, *j* = 1,2,…, *n* and *i* ≠ *j*, *t* = 0,1,…, *n*), then the sequence is *m*_1_, *m*_2_,…, *m*_*n*_ where (*m*_*k*_, *k* = 1,2,…, *n*) are the setup numbers of the same machine. Finally, (*m*_*k*_, *m*_*k*+1_) are compared using the following equations (where *k* = 1,2,…, *n* − 1):

(10) $$\begin{array}{c} P_{\left(k,k+1\right)}^{t}=\{\begin{array}{cc} 1, & m_{k}\neq m_{k+1},\\ 0, & m_{k}=m_{k+1}. \end{array} \end{array}$$

The  total  setup  number  for  each  machine  is

(11) $$\begin{array}{c} t=\sum_{k=1}^{n}P_{\left(k,k+1\right)}^{t}+1. \end{array}$$

The evaluation setup number (E.S.N) is determined for all machines using

(12) $$\begin{array}{c} \text{E}.\text{S}.\text{N}=\sum_{t=0}^{n}\left(\sum_{k=1}^{n}P_{\left(k,k+1\right)}^{t}+1\right). \end{array}$$

Finally, the total fitness values of the efficient frontiers are calculated based on these two objectives [27]. This step is repeated for each possible chromosome (*X*_*i*_) in the population size.

### 3.3. Adaptive Genetic Operators

#### 3.3.1. Fuzzy Expert Experience Controller

Fuzzy expert experience controller (FEEC) is implemented to set the population size, number of generations, and other variables prior to execution of the GA. The FEEC is used to control the parameters automatically. The controller comprises four principal components, listed as follows:

1. fuzzification interface, which converts crisp input data into suitable linguistic values;
2. fuzzy rule base, which consists of a set of linguistic control rules incorporating heuristics that are used to achieve a faster rate of convergence;
3. fuzzy inference engine, which is a decision-making logic that employs rules of the fuzzy rule base to infer fuzzy control actions in response to fuzzed inputs;
4. defuzzificationinterface, which yields a crisp control action from an inferred fuzzy control action.

The first step (fuzzification) involves defining the membership function of inputs and outputs for each parameter. The properties of these attributes (i.e., fuzzy variables, fuzzy set and fuzzy number of each input, and output variable) are listed in Table 2. The membership functions of the fuzzy input and output variables for each aspect are formulated based on the knowledge and experience of GA experts, as well as related literature [32, 36]. The membership functions are defined by the population size, ranging from 10 to 160 in the experiments. The membership function variables are all trapeziums, ranging from 0 to 1 on the defined universe of discourse. The inputs and outputs and their associated sets of linguistic labels are illustrated in Table 2. The input variable is chromosome size (CS) and is divided into four trapezoid membership functions, namely, “very short (VSH)”, “short (SH)”, “long (LO),” and “very long (VL).” The membership values assigned to the two output variables are “population size (PS)” and “number of generations (NG).” Four linguistic labels, namely, “small (S),” “medium (M),” “big (B),” and “very big (VB)” are used to represent Output 1 (i.e., population size (PS)) in which the universe of discourse has values within the range of 0 and 200. Likewise, the same four linguistic labels are used to represent output 2 (i.e., number of generations) whereby the universe of discourse consists of values ranging between 0 and 1750.

The second step involves formulating the fuzzy rules for each trapezoidal membership function. The number of rules (*N*) required to control the system is given by

(13) $$\begin{array}{c} N=\sum_{j=1}^{m}\left(\prod_{i=1}^{n}L_{i}\right), \end{array}$$

where *m* represents the number of sets of rules, *L*_*i*_ represents the number of membership functions or levels, and *N* represents the number of input variables used in one set of rules. If *M* = 1, *N* = 1, and *L*_*i*_ = 4, the number of rules (*N*) will be 1 × 1 × 4 = 4. The relationship between the fuzzy input and fuzzy output variables for each station is developed using the Mamdani fuzzy IF-THEN MIN-MAX rules. The fuzzy rules are developed based on interviews with experienced experts. The collection of fuzzy rules approximately represents the human thinking process during decision making. In single-input multiple-output (SIMO) systems, these rules are considered to be heuristic design rules of the following form [48].

IF input (1) is “A,” then output (1) is “B” and output (2) is “C.”

A sample of the generated fuzzy rules for chromosome size (CS) input variable is presented in Table 3.

The basic fuzzy rule-based system used for the fuzzy population and generation size model is summarized in Table 4. It can be seen from Table 4 that the Mamdani fuzzy inference model is used as the conjunction operator and is based on the aggregation function maximum (Max).

The third step involves designing fuzzy implication (FI) for each rule. The rules' weights need to be determined prior to FI. The input of the implication process is a single number given by the antecedent, and the output is a fuzzy set. Aggregation is equivalent to fuzzification, when there is only one input to the controller. The output decisions are based on testing all rules in the FEEC, and the rules (*R*_*i*_) must be combined in a specific manner for decision making. The aggregation operation is used during the calculation of the degree of aggregation (*a*_*i*_) for the condition of a rule (*R*_*i*_). Aggregation is a process by which the fuzzy sets that represent the outputs of each rule are combined into a single fuzzy set. The input of the aggregation process is the list of truncated output functions returned by the implication process for each rule. 

After implication, the output decisions following testing of all rules in the FEEC and the rules (*R*_*i*_) must also be combined in a specific manner for decision making. The rules are placed to show how the output of each rule is combined or aggregated into a single fuzzy set whose membership function assigns a weight for every output (tip) value. Finally, these fuzzy outputs need to be converted into a scalar output quantity so that the nature of the action to be performed can be determined by the centre of gravity method. The most popular defuzzification method is the centroid calculation method, which returns the centre of gravity (COG) under the curve. This technique was developed by Sugeno in 1985 and is the most commonly used technique as well as being highly accurate. The centered defuzzification technique can be expressed as

(14) $$\begin{array}{c} X^{*}=\frac{\int \mu_{i}\left(x\right)x\;dx}{\int \mu_{i}\left(x\right)dx}, \end{array}$$

where X* is the defuzzified output, *μ*_*i*_(*x*) is the aggregated membership function, and *x* is the output variable. The only disadvantage of this method is that it is computationally difficult for complex membership functions.

#### 3.3.2. Fuzzy Automatic Learning Controller

**Figure 3:**
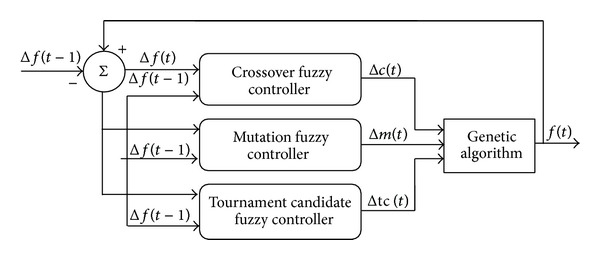
Block diagram of proposed fuzzy controlled genetic algorithm for tournament candidate and crossover and mutation rates.

The fuzzy automatic learning controller (FALC) technique is implemented to adjust the GA operators (e.g., tournament candidate selection and crossover and mutation rates) automatically during the optimization process. The heuristic updating principles of the tournament candidate and crossover and mutation rates are such that the change in the average fitness of the population is greater than zero and remains the same sign in consecutive generations. Put simply, the GA operators will increase and vice versa. The block diagram of the proposed fuzzy logic controlled genetic algorithm (FLCGA) for tournament candidate and crossover and mutation rates is shown in Figure 3. The tournament candidate and crossover and mutation rates can be adjusted adaptively or dynamically during the evolution process due to the fuzzy logic embedded in the genetic operators. 

From Figure 3, the online FLCs are used to adapt the tournament candidate and crossover and mutation rates with the aim of improving the convergence rate significantly. The FLCs shown in Figure 3 are used to control the parameters automatically and comprise four principal components, which are listed as follows:

1. fuzzification interface, which converts crisp input data into suitable linguistic values;
2. fuzzy rule base, which consists of a set of linguistic control rules incorporating heuristics used to achieve a faster convergence rate;
3. fuzzy inference engine, which is a decision making logic that employs rules from the fuzzy rule base to infer fuzzy control actions in response to the fuzzed inputs;
4. defuzzificationinterface, which yields a crisp control action from an inferred fuzzy control action.

The fundamental concept of Song et al. [37] is implemented to regulate the GA operators (tournament candidate “Δ*tc*(*t*),” crossover rate “Δ*c*(*t*),” and mutation rate “Δ*m*(*t*)”) adaptively using FLC during the genetic search process. The heuristic updating strategy for these three operators is based on the changes in the average fitness of the GA population for two continuous generations, “Δ*f*(*t*)” and “Δ*f*(*t* − 1).” The number of tournament candidates (TC), crossover rates (*C*), mutation rates (*M*), and occurrence rates of the operators will increase if they consistently produce better offspring during the recombination process. Likewise, the number of TC,* C*,* M*, and occurrence rate of the operators will decrease if they consistently produce poorer offspring. This scheme is based on the principle that well-performing operators are encouraged to produce a higher number of offspring, while reducing the chance for poor-performing operators to destroy the potential individuals during the recombination process. The inputs of the fuzzy tournament candidate and crossover and mutation rates are changes in fitness at two consecutive steps (i.e., Δ*f*_avg_(*t* − 1) and Δ*f*_avg_(*t*)) while the outputs are the changes in the number of tournament candidates (Δ*tc*(*t*)) and crossover rates (Δ*c*(*t*)) and mutation rates “Δ*m*(*t*).” The change in average fitness at generation (*t*) for the minimization problem (i.e., Δ*f*_avg_(*t*)) is set using

**Figure 4:**
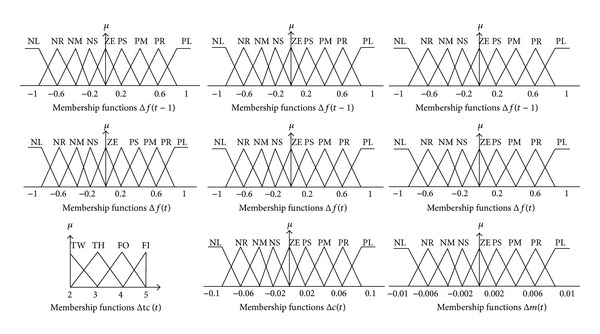
Membership functions of inputs (Δ*f*(*t* − 1)  and  Δ*f*(*t*)) and outputs (Δ*tc*(*t*), Δ*c*(*t*), and  Δ*m*(*t*)).

(15) Δfavg(t)=(fpar−size−(t)−foff⁡−size−(t))×λ=(∑k=1par−sizefk(t)par−size−∑k=par−size+1par−size+off⁡−sizefk(t)off⁡−size)×λ,

where *k* is a scaling factor to normalize the average fitness values for defuzzification in the FLC and is varied accordingly to the given problem. The scaling factor *k* is required to normalize the average fitness values. The regulation and procedure of Δ*tc*(*t*), Δ*c*(*t*), and Δ*m*(*t*) begin with the application of Δ*f*_avg_(*t* − 1) and Δ*f*_avg_(*t*) based on the average fitness values, as in Algorithm 1, where *ε* is a given real number in the proximity of zero and *γ* and −*γ* represent the given maximum and minimum values, respectively, for the fuzzy membership function of the fuzzy input and output linguistic variables, as illustrated in Figure 4. The labels of the membership function are as follows: NL: negative larger, NR: negative large, NM: negative medium, NS: negative small, ZE: zero, PS: positive small, PM: positive medium, PR: positive large, PL: positive larger, TW: two, TH: three, FO: four, and FI: five.

The Δ*f*_avg_(*t* − 1) and Δ*f*_avg_(*t*) values are normalized correspondingly within the range of [−1.0,1.0]. The Δ*tc*(*t*) values are normalized within the range of [2,5], whereas the Δ*c*(*t*) and Δ*m*(*t*) values are normalized within the range of [−0.1,0.1] and [−0.01,0.01], respectively, depending on their corresponding maximum values. 

The application of a new tournament candidate fuzzy decision table designed based on conventional design concepts as well as the fuzzy decision table for crossover and mutation rates is given in Table 5. 

For simplicity, a look-up table for the control actions of the tournament candidate and crossover rate and mutation rate FLCs is shown in Table 6. The quantified levels corresponding to the linguistic values of input and output fuzzy variables of the tournament candidate and crossover and mutation rates FLCs are designated as −4, −3, −2, −1, 0, 1, 2, 3, and 4, respectively. In order to calculate the crossover and mutation rates based on (2), let *x* represent the quantified levels of Δ*f*(*t* − 1), *y* the quantified levels of Δ*f*(*t*), and *z* the quantified levels of Δ*c*(*t*) and Δ*m*(*t*). Equation (16), which is illustrated in Table 6, is given by

(16) $$\begin{array}{c} z\leq \alpha x+\left(1-\alpha \right)y, \end{array}$$

where *x* and *y* represent the first and second inputs of the average fitness functions (Δ*f*(*t* − 1)) and Δ*f*(*t*)), respectively, whereas *z* is a minimum integer which is less than *ax* + (1 − *α*)*y*, and *a* is an adaptive coefficient which varies with the fitness value of the whole population. It is found that the crossover and mutation rates FLCs yield good performance when *α* equals 0.5.

**Figure 5:**
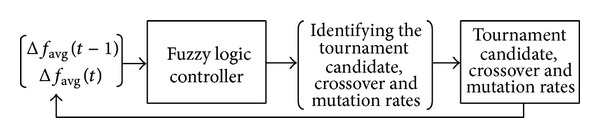
Regulating strategy of the fuzzy logic controller in the genetic algorithm loop.

Fuzzy inference engine, which is a decision making logic, employs the rules of the fuzzy rule base to infer fuzzy control actions in order to generate fuzzy outputs based on the inputs. The input values are assigned to the indices *x* and *y* upon identification of the fitness function values (Δ*f*_avg_(*t* − 1) and Δ*f*_avg_(*t*)). The input values correspond to the controller actions based on the fuzzy decision table in order to identify the values of the tournament candidate, crossover rate, and mutation rate, as shown in Table 5. For fuzzy inference, the regulating strategy of the FLCs in the GA loop is shown in Figure 5.

The outputs of the tournament candidate (Δ*tc*(*t*)) and changes in the FLC for crossover and mutation rates (Δ*c*(*t*) and Δ*m*(*t*)) are generated upon identification of *Z*(*x*, *y*) for the tournament candidate and crossover and mutation rates using Table 6 and (17), (18), and (19), respectively:

(17) $$\begin{array}{c} \Delta tc\left(t\right)=Z\left(x,y\right), \end{array}$$

(18) $$\begin{array}{c} \Delta c\left(t\right)=Z\left(x,y\right)\times 0.02, \end{array}$$

(19) $$\begin{array}{c} \Delta m\left(t\right)=Z\left(x,y\right)\times 0.002, \end{array}$$

where *Z*(*x*, *y*) consists of the corresponding values of Δ*f*_avg_(*t* − 1) and Δ*f*_avg_(*t*) for defuzzification from Table 6, in which *x*, *y* ∈ {−4, −3, −2, −1,0, 1,2, 3,4}. The values 0.02 and 0.002 are selected in order to regulate the increasing and decreasing range of rates for the crossover and mutation operators, respectively. The tournament candidate is computed using

(20) $$\begin{array}{c} tc\left(t\right)=\Delta tc\left(t\right). \end{array}$$

The rate of the crossover and mutation operator is updated using (21) and (22), respectively, once the changes of the crossover rate (Δ*c*(*t*)) and mutation rate (Δ*m*(*t*)) have been identified using (5) and (6):

(21) $$\begin{array}{c} P_{c}\left(t\right)=P_{c}\left(t-1\right)+\Delta c\left(t\right), \end{array}$$

(22) $$\begin{array}{c} P_{m}\left(t\right)=P_{m}\left(t-1\right)+\Delta m\left(t\right). \end{array}$$

The adjusted rates should not exceed the range of 0.5–1.0 and 0.0–0.1 for *P*_*c*_(*t*) and *P*_*m*_(*t*), respectively [33].

Tournament selection is carried out once the adaptive genetic operators (i.e., number of selected candidates and crossover and mutation rates) have been identified. Tournament selection is a method used to reproduce a new generation which is proportional to the fitness of each individual. The main characteristics of tournament selection are summarized as follows.

- Tournament selection is quite useful in certain situations, such as multiobjective optimization.
- Tournament selection uses only local information.
- Tournament selection is easily implemented with low time complexity.
- Tournament selection can be easily implemented in a parallel environment.

However, tournament selection also suffers from selection bias, which means that the best one will not be selected if it is very unlucky.

Following this, crossover probability is used, which crosses over parents to form new offspring (children). In the crossover phase, all chromosomes (except for the elite chromosome) are paired up and crossed over with a probability crossover rate. Crossover is accomplished by choosing a site randomly along the length of the chromosome and exchanging the genes of two chromosomes (parents) for each gene past this crossover site. The details for crossover are given as follows.

(1) Identify the number of different requirements for manufactured products (P. No).
(2) Create the one random number between product type numbers (P. No):
   for (int *i* = 0; *i* < P.No; *i*++)
   
{
     Product Random = rand( ) % Product;
   
}
(3) Identify the gene code number (*X*_*i*_) for the selected product obtained from the previous step.
(4) Search and identify the gene code from parent A based on random product selection and transfer these gene codes to child B, which is exactly at the same gene location.
(5) Search and identify the gene code from parent B based on random product selection and transfer the gene code to child A, which is exactly at the same gene location.
(6) Transfer the remaining gene code from parent A to the gene blank of child A.
(7) Transfer the remaining gene code from parent B to the gene blank of child B.
(8) Calculate the number of crossovers based on the crossover rate (*P*_*c*_(*t*)) from (21) and population size (PS):
  (23) $$\begin{array}{c} \text{Number}\;\;\text{of}\;\;\text{crossovers}=\frac{P_{c}\left(t\right)\times \text{PS}}{2}. \end{array}$$
(9) Once new offsprings have been created, the new offspring will have previous chromosomes in the current generation.

In crossover operation, the worst or weakest chromosomes will fade away whereas the characteristics of the chromosomes will change continuously during mutation operation. The elite chromosome will not be subjected to mutation in the next generation. Consequently, GA does not lead to annihilation since several chromosomes (one, two, or three) from each generation are transferred directly to the next generation. Mutation is not applied on chromosomes which are immune. It is possible to maintain a fixed fitness value in some generations, but they will never deteriorate. The number of elitism is assigned as three.

Following crossover operation, the genes will mutate to any one of the codes at a mutation rate for each gene in the chromosomes, with the exception of the elite chromosome. When the crossover and mutation operations are complete, the chromosomes will be evaluated for another round of selection and reproduction. Considering elitism and after identifying the parts in which mutation will be applied, the number of mutations in each generation is calculated using (24) based on the mutation rate (*P*_*m*_(*t*)), population size (PS), and maximum gene code (Max. *X*_*i*_):

(24) $$\begin{array}{c} \text{Number}\;\;\text{of}\;\;\text{ mutations }≅\left[\left(\text{PS}\times \text{Max.}{\text{X}}_{i}\right)\times P_{m}\right]. \end{array}$$

The swap mutation operator is used in this study, in which two positions are selected at random and their contents are swapped as a general procedure. After identifying the number of gene mutations, a set of rules need to be devised for the mutation of genes from point A to B and vice versa, while focusing on the stability of the chromosome sequence. It is important that the sequence of the chromosomes is not displaced. There are eight sets of rules for this step which are classified into two groups, as follows.

1. Four rules are used to check the mutation based on the part sequence (Rules 1 and 2) and product assembly (Rules 3 and 4) of genes from A to B.
2. Four rules are used to check the mutation based on the part sequence (Rules 5 and 6) and product assembly (Rules 7 and 8) of genes from B to A.

It shall be noted that the position of A is before B. The eight mutation rules should be checked thoroughly to ensure their ideal applications. In the adverse case, the eight rules ought to be done over.

Having performed crossover, elitism and mutation operations, the most ideal chromosomes of the current generation, are compared and evaluated to identify its total value, after checking its termination in the following step. The loop of chromosome generations is terminated when certain conditions are met. The elite chromosome is returned as the best solution once the termination criteria are met. The termination criteria are listed below.

(1) If the number of generations reaches its maximum, the loop of chromosome generations is terminated.
(2) If there are no changes in the elite solution (i.e., no changes in fitness function value), the loop of chromosome generations is terminated using
  (25) $$\begin{array}{c} \text{Fitness}\;\;\text{Value}\;\;\left(X_{i}\right)-\text{Fitness}\;\;\text{Value}\;\;\left(X_{i+1}\right)\leq 0.0001. \end{array}$$

## 4. Computational Experiments

### 4.1. Model Development

In this section, the results of multiobjective fuzzy mixed assembly line sequencing model are presented based on the development of new test-beds. According to Silberholz and Golden [49], new test-beds are developed when an existing test-bed is insufficient. There are two points which need to be addressed when developing new test-beds, that is, the purpose of developing the test-beds as well as the accessibility of new test instances [49]. The purpose of a problem suite is to emulate real-world problem instances with a variety of test cases and difficulty levels. When creating a new test-bed, the focus is to provide others with access to problem instances. This enables other researchers to make comparisons easily, while ensuring that the problem instances are widely used. One way to ensure this is to create a simple generating function for the problem instances. Capturing the real aspects of a problem is particularly significant when developing a new test-bed. 

#### 4.1.1. Input Data

A hypothetical numerical test-bed example is designed to test the fuzzy mixed-model assembly line sequencing problem. The input data of the hypothetical numerical example are given in Table 7, consisting of 50 jobs and 20 parts in order to produce four products. There are five machine tools (one lathe, two CNC, and two robots) assigned to assemble four products.

#### 4.1.2. Initialization of Parameters and Fuzzy Variables

The initialization of parameters for the mixed-model assembly line sequencing example is shown in Table 8. It can be seen that 50 jobs (*J*_*i*_, *i* = 1,2,…, 50) are required to produce 20 parts (*P*_*j*_, *j* = 0,1,…, 19) and these parts are assembled to produce four types of products (P.No_*k*_, *k* = 1,2, 3,4). The number of tool changes is 5 (labelled as A, B, C, D, and E) and these tools are assigned to five machines (*M*(*X*_*i*_), *i* = 0,1, 2,3, 4). The job sequence is dependent upon the part and product assembly and is described as follows. First, the job number is assigned to produce the first product, ranging from 1 to 10 (P.No_1_, *J*_*i*_ = 1,2,…, 10). It shall be highlighted that there are 10 jobs in this case and they are sequenced to produce three parts according to the following order. Job numbers 1, 2, and 3 are assigned to produce Part_0_  ((*P*_*i*_), *i* = 0 → *J*_1_, *J*_2_, *J*_3_), while job numbers 4 and 5 are assigned to produce Part_1_  ((*P*_*i*_), *i* = 1 → *J*_4_, *J*_5_). Job numbers 6 and 7 are assigned to produce Part_2_  ((*P*_*i*_), *i* = 2 → *J*_6_, *J*_7_), whereas job numbers 8, 9, and 10 are assigned to produce Part_3_, which is a subpart of the product assembly ((*P*_*i*_), *i* = 3 → *J*_8_, *J*_9_, *J*_10_). Production of the second, third, and fourth products is based on the sequence described for the first product, as shown in Table 8. The fuzzy processing time of each job (*J*_*i*_, *i* = 1,2,…, 10) is defined as a triplet (*a*_1_, *a*_2_, *a*_3_). The total operating time is based on the fuzzy triangular time, and is required to complete the jobs sequentially when producing each part, in which each part is assigned to a machine (*M*_*i*_, *i* = 0,1, 2,3, 4). The total operating time is defined as (${\tilde{\text{OP}\cdot P}}_{i}(a_{1},a_{2},a_{3}),\;\;i=0,1,\ldots ,19;\;\;a$ = time). The total travelling time based on the above information is defined as (${\tilde{\text{T}.\text{t}\cdot P}}_{j}(a_{1},a_{2},a_{3}),\;\;j=0,1,\ldots ,19;\;\;a=\text{time}$). The operation and travelling time is fuzzy numbers, which are indicated by *a*_1_, *a*_2_, and *a*_3_. The parameters *a*_1_, *a*_2_, and *a*_3_ represent the optimistic time, normal time, and pessimistic time, respectively.

**Figure 6:**
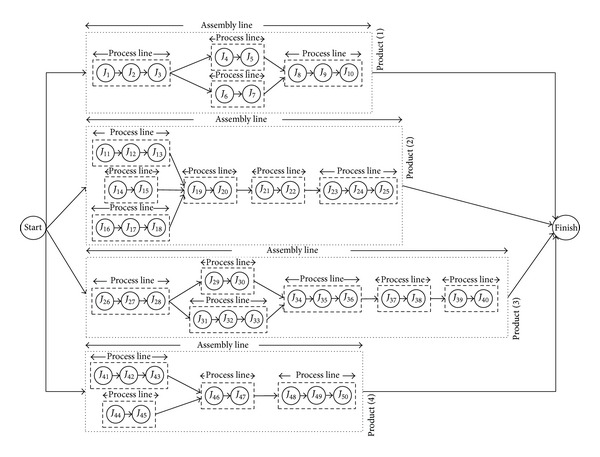
Example of mixed-model assembly line sequencing (50 jobs, 20 parts, and 4 products).

**Figure 7:**
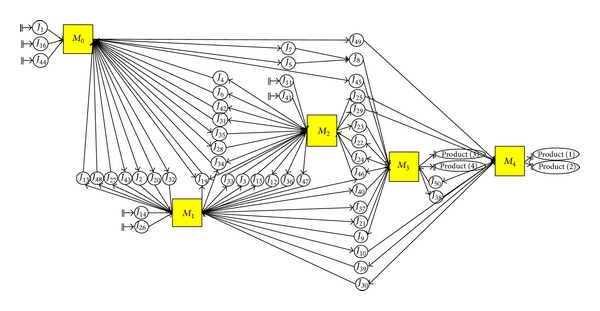
Assignment of parts to their respective machines based on the example for mixed-model assembly line sequencing.

The development of a model for mixed-model assembly line sequencing is presented in Figure 6, based on the parameters listed in Table 8. The solid arrows (→) represent the order of the product line (sequence of part production), discrete part manufacturing assembly, leading to the finished products, as indicated by the job numbers. Suppose that the production process involves manufacturing four products using the same assembly line. In other words, four different products are manufactured simultaneously on the assembly line, and hence the problem is a mixed-model assembly line problem. In this example, 20 parts need to be manufactured using five machines. From Figure 6, the order of the production of parts is represented by the dashed rectangles and is termed as the process line. The assembly lines are represented by the dotted rectangles. The assignment of parts to their respective machines based on job number is illustrated in Figure 7.

#### 4.1.3. Multiobjective Evaluation

The final results based on the existing and optimized data are shown in Table 9. The overall results show that the existing fuzzy data is improved by optimization. The total fuzzy existing scheduling time is optimized from (166, 250, 266) to (62.5, 73, 88.5). The total fuzzy setup numbers for the existing and optimized data are (44, 44, 44) and (37, 37, 39), respectively. The total fuzzy existing efficient frontier is (105, 147, 155), while the optimized one is (49.25, 54.5, 63.75). The total fuzzy existing scheduling and setup time are optimized from (254, 338, 354) to (135, 145, 168). The total fuzzy operation setup time for the existing and optimized data are (88, 88, 88) and (72, 72, 78), respectively. The total fuzzy existing changing setup cost is reduced from ($3520, $3520, $3520) to ($2880, $2880, $3120). The total fuzzy unit produced per day for the existing and optimized data is approximately (1.80, 1.92, 2.89) and (5.33, 6.58, 7.62), respectively. The total existing fuzzy percentage efficiency is optimized from (10.32%, 10.90%, 11.45%) to (30.16%, 32.22%, 35.34%).

### 4.2. Method Development

**Figure 8:**
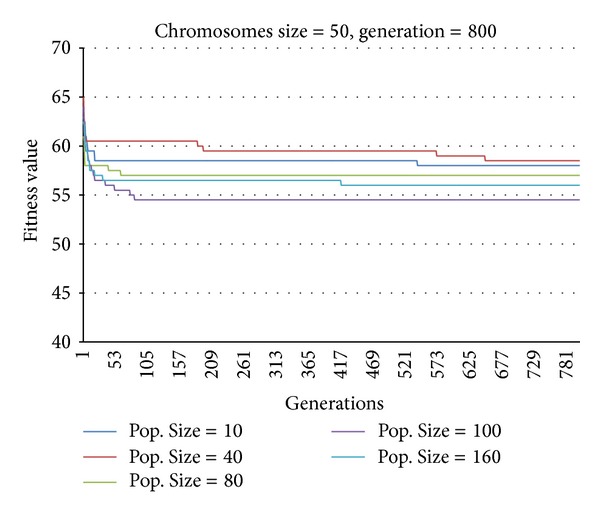
Fitness values versus number of generations for a chromosome size of 50.

The overall results obtained from the dynamic Fuzzy GA method are presented in this section, based on the hypothetical numerical example (test-beds) described in Section 4.1. The optimum fuzzy population size and generation size, as well as the selection of a suitable tournament candidate, crossover rate, and mutation rate, are discussed. The results obtained from both GA and fuzzy GA methods are also compared and discussed in detail in this section. The fitness values generated based on the fuzzy rule base with respect to the number of generations and population size are shown in Figure 8. The purpose of the comparative analysis is to select the best population size and number of generations, based on the job number (chromosome size) of the mixed-model assembly line sequencing problem. Figure 8 shows the fitness values for a chromosome size of 50, which indicates that 50 jobs are required to produce four products with a 19-part assembly assigned to five machines. It is found that the fitness value is 58 when the population size is 10 and the number of generations is 539. The fitness value increases slightly with a value of 59 when the population size and the number of generations is 40 and 652, respectively. The fitness values are determined to be 58 and 57 for a population size of 80 and 160, and the corresponding number of generations is 61 and 416, respectively. The results show that for a chromosome size of 50, the highest fitness value is found to be 54.5 when the population size is 100 and the number of generations is 83.

**Figure 9:**
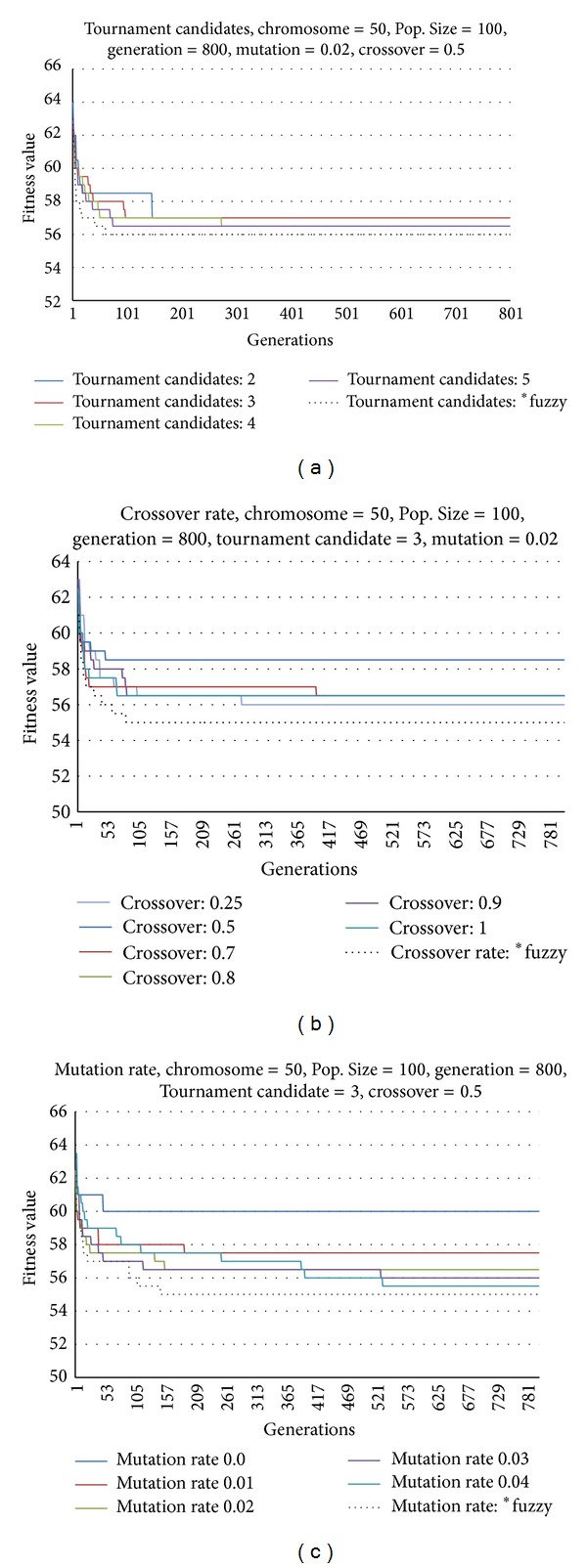
Comparison between tournament candidates and crossover and mutation rates.

The results of the tournament candidate, crossover rate, and mutation rate for a mixed-model line sequencing problem with 50 chromosomes (50 jobs), fixed population size (of 100), and 800 generations are presented in Figure 9. The results are compared with the dynamic fuzzy tournament candidate and crossover and mutation rates. A comparison between the performance of the fixed tournament candidate (mutation rate: 0.02 and crossover rate: 0.5) and dynamic fuzzy tournament candidate (in which the tournament candidate is varied from 2 to 5) is shown in Figure 9(a). The results show that the selection of tournament candidates affects the performance of the GA. It can be observed that a decrease in the number of tournament candidates improves the convergence of the GA at the expense of reduced accuracy. A comparison between the performance of the GA having a fixed crossover rate (tournament candidate: 3 and mutation rate: 0.02) and dynamic fuzzy crossover rate (whereby the crossover rate is changed from 0.25 to 1) is shown in Figure 9(b). Similarly, it can be seen that changes in the crossover rate have a significant influence on the results, whereby a high crossover rate yields faster convergence with reduced accuracy. From Figure 9(b), a crossover rate of 1 yields faster convergence compared to a crossover rate of 0.9. However, a crossover rate of 0.25 offers higher accuracy. A comparison between the performance of the GA with fixed mutation rate (tournament candidate: 3 and crossover rate: 0.5) and dynamic fuzzy mutation rate (in which the mutation rate is varied from 0.0 to 0.04) is shown in Figure 9(c). It is also evident that the mutation rate has a significant influence on the performance of the GA. It can be noted that a lower mutation rate yields faster convergence at the expense of reduced accuracy. From Figure 9(c), it can be seen that a mutation rate of 0.01 gives faster convergence compared to a mutation rate of 0.04; however, the latter mutation rate offers higher accuracy. The most significant finding of this study is that the dynamic fuzzy tournament candidate, crossover rate, and mutation rate yield faster convergence with higher accuracy in the optimum fitness values.

The optimum fitness values with respect to the number of generations and time taken to achieve convergence for various tournament candidates, crossover rates, and mutation rates are summarized in Table 10. From Table 10, the fastest convergence is achieved when the number of generations is 60, whereas the optimum fitness value is more accurate when the number of generations is 56 for the dynamic fuzzy tournament candidate. The lowest fuzzy GA execution time is 1.45 s. It can be observed from Table 10 that faster convergence is attained for the dynamic fuzzy crossover and mutation rates when the number of generations is 79 and 147, respectively. However, the optimum fitness values for both crossover and mutation rates have a higher accuracy when the number of generations is 55. The lowest fuzzy GA execution time is found to be 2.50 and 3.82 s, respectively.

**Figure 10:**
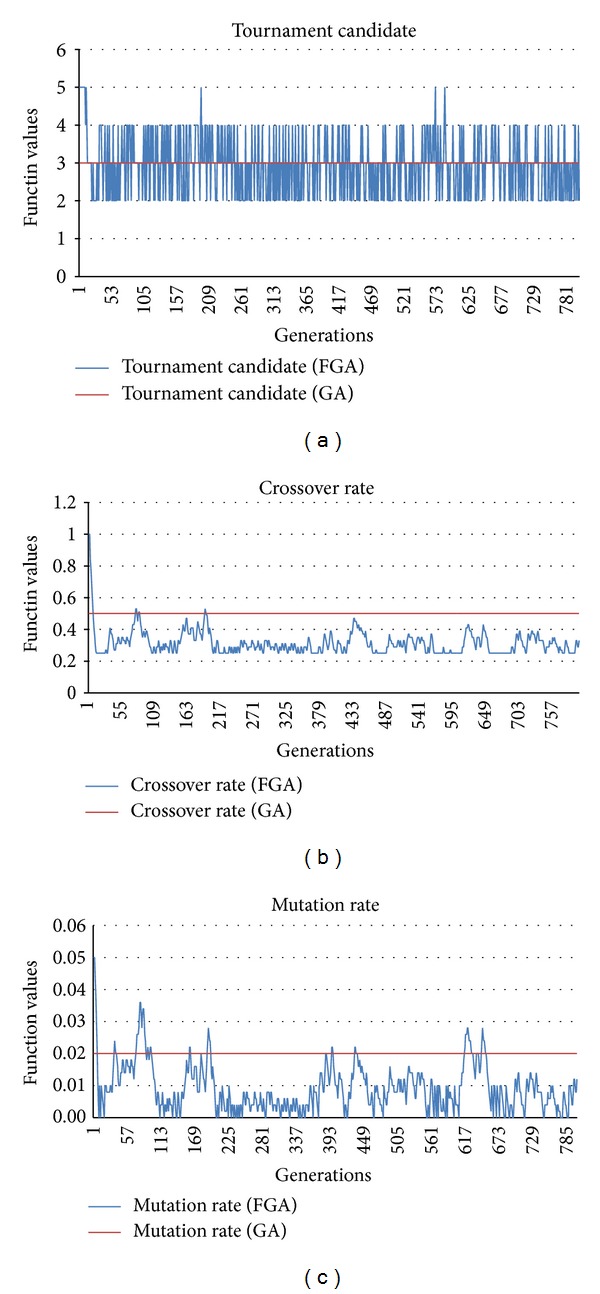
Comparison of behavior between fixed and dynamic fuzzy tournament candidates and crossover and mutation rates.

A comparison of the static and dynamic behaviors of the tournament candidate, crossover rate, and mutation rate between conventional GA and FGA is shown in Figure 10. The conventional GA is used for solving the mixed-model assembly line sequencing problem and comprises the following parameters (chromosome size: 50, population size: 100, number of generations: 800, tournament candidate: 3, crossover rate: 0.5, and mutation rate: 0.02). The dynamic FGA is also implemented for solving the above problem using the same chromosome size, population size, and number of generations as for the conventional GA. However, it shall be emphasized that fuzzy tournament candidate, fuzzy crossover rate, and fuzzy mutation rate are used as the parameters for dynamic FGA. The results show that the tournament candidate, crossover rate, and mutation rate are dynamically and automatically modified during the optimization process using the fuzzy logic controller.

**Figure 11:**
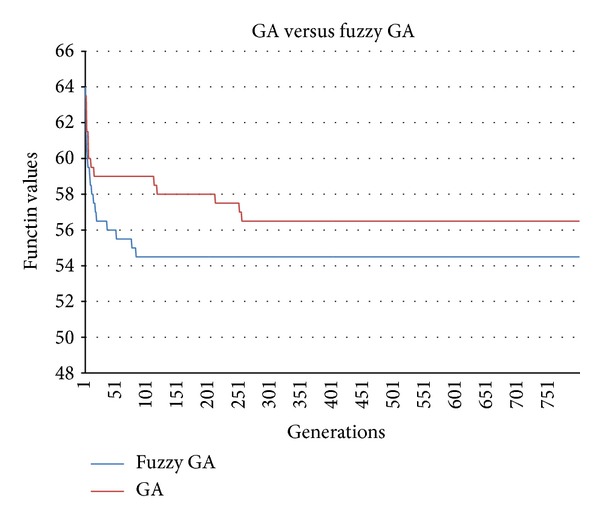
Final results of GA versus FGA.

Figure 11 and Table 11 show the final results of the dynamic FGA and conventional GA based on the optimum fitness value and number of generations. The FGA designed with three fuzzy dynamic parameter controllers (i.e., tournament candidate, crossover rate, and mutation rate) exhibits a superior performance compared to the existing GA. The results reveal that the FGA is capable of rapid and efficient searching compared to the standard GA for solving the mixed-model assembly line sequencing problem. It is evident from the results that the optimum fitness value for dynamic FGA (54.5) is more accurate than the conventional GA (57.5). The FGA yields faster convergence, whereby the number of generations is 87. In contrast, convergence is achieved only when the number of generations is 243 for conventional GA. The FGA also gives a lower execution time of 2.15 s compared to the GA, which has an execution time of 6.31 s.

## 5. Conclusion

It is known that mixed-model assembly line sequencing is a problem with multiple conflicting objectives. A mixed-model assembly line sequencing optimization model is developed in order to address two conflicting objectives, namely, minimizing the make-span (i.e., minimizing scheduling time, travelling time, and machine idle time and maximizing production) and minimizing the setup time (i.e., minimizing the number of machine setup tool change and minimizing the machine setup cost) simultaneously, which occur when switching between different products. These objectives have been achieved successfully and tested using a hypothetical numerical test-bed. The hypothetical numerical test-bed involves 50 jobs to produce 20 parts using five machines in order to assemble four products. Triangular and trapezoidal fuzzy numbers are applied to the operation time and travelling time variables. The fuzzy numbers are categorized as optimistic, medium, and pessimistic fuzzy total scheduling time. The results show that the fuzzy total scheduling time (166, 250, and 266) decreases to (62.5, 73, and 88.5) after optimization. Comparison is made between the existing and optimized results representing the efficiency and idle time of each machine. The existing and optimized results of the total scheduling time, total setup number, total efficient frontier, total scheduling time with setup time, total operation setup time, total changing setup cost ($), and total number of units produced per day are also compared.

In this study, a dynamic fuzzy GA approach is proposed, in which a fuzzy expert experience controller (FEEC) and automatic learning dynamic fuzzy controller (ALDFC) are integrated with genetic algorithm in order to solve a multiobjective mixed-model assembly line sequencing problem. The aim of developing this method is to enhance the performance and effectiveness of GA. The fuzzy expert experience controller is used in order to decide the number of generations and population size in the GA. In order to dynamically control the genetic operators, three automatic learning dynamic fuzzy controllers are implemented to select the number of tournament candidates and set the crossover and mutation rates based on existing conditions. The performance of the FGA has been evaluated with respect to the adaptive control parameters. The results show that the FGA exhibits superior performance compared to the conventional GA. The FGA is capable of searching faster and more efficiently compared to the standard GA when solving the mixed-model assembly line sequencing problem due to the following reasons.

1. The population size and control parameters can be chosen appropriately for the problem under investigation.
2. The control parameters can be adjusted on-line to adapt dynamically to new situations.
3. The FGA can assist users in accessing, designing, implementing, and validating genetic algorithms for a given task.

The optimal fitness value for the dynamic FGA (54.5) is more accurate compared to that for conventional GA (57.5). The FGA attains faster convergence (number of generations: 87) whereas the conventional GA achieves convergence when the number of generations is 243. The FGA also yields a lower execution time (2.15 s) compared to the conventional GA (6.31 s).

## Figures and Tables

**Algorithm 1:**
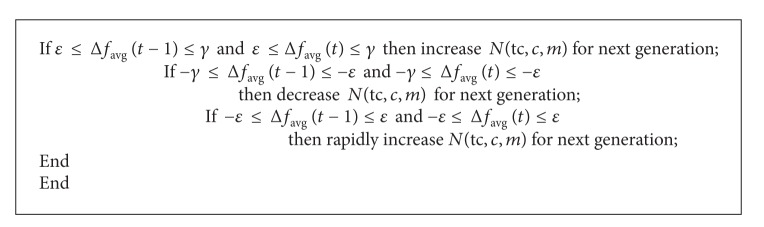


**Table 1:** Parameters used to develop the mixed-model assembly line sequencing model with genetic algorithm.

Gene code: (*X*_*i*_, *i* = 1, 2, …, *n*)	Machine number: (*M*_*i*_, *i* = 1, 2, …, *n*)	Tournament candidate: (TC)
Jobs: (*J*_*i*_, *i* = 1, 2, …, *n*)	Fuzzy operation time: $\left(\tilde{\text{OP}}(a_{n},b_{n},c_{n})\right)$	Crossover rate: (CR)
Product number: (P.NO_*i*_, *i* = 1, 2, …, *n*)	Fuzzy traveling time: $\left(\tilde{\text{T.}\;\text{t}}(a_{n},b_{n},c_{n})\right)$	Mutation rate: (MR)
Assembly sequence: (A.S_*i*_, *i* = 0,1,…, *n*)	Elitism: (*E* = 3)	Fuzzy start time: ($\tilde{\text{S.t}}$)
Setup number: (S.No = A, B,…, Z)	Population size: (PS)	Fuzzy total scheduling time: ($\tilde{C}$)
Part: (*P*_*i*_, *i* = 1, 2, …, *n*)	Maximum generations: (MaxG)	Chromosome: (Ω)

**Table 2:** List of properties of attributes.

Fuzzy variables	Fuzzy set	Fuzzy number
Fuzzy input variables		
Chromosome size (CS)	VSH, SH, LO, VL	(0,0, 10,20)(10,15,25,30)(20,30,50,70)(50,70,130,130)
Fuzzy output variables		
(i) Population size (PS)	S, M, B, VB	(0,20,60,80)(60,70,90,100)(80,95,105,120)(110,130,200,200)
(ii) Number of generations (NG)	S, M, B, VB	(0,50,150,200)(150,200,400,450)(400,450,1150,1200)(110,1200,1700,1750)

**Table 3:** Fuzzy IF-THEN rules for chromosome size (CS) input variable.

Rule number	Fuzzy input variable	Fuzzy output variables	
	Chromosome size (CS)	Population size (PS)	Number of generations (NG)
1	Very short (VSH)	Small (S)	Small (S)
2	Short (SH)	Medium (M)	Medium (M)
3	Long (LO)	Big (B)	Big (B)
4	Very long (VL)	Very big (VB)	Very big (VB)

**Table 4:** Summary of the basic fuzzy rule-based system.

Aspect number	Aspect name	Number of inputs	Number of outputs	Number of rules	Aggregation operator	Inference model
1	Population and generation size	1	2	4	Max	Mamdani

**Table:** (a) Δtc(*t*)

Δ*f*(*t*)	Δ*f*(*t* − 1)								
	NL	NR	NM	NS	ZE	PS	PM	PR	PL
NL	FI	FI	FI	FO	FO	TH	TH	TW	TW
NR	FI	FI	FO	FO	TH	TH	TW	TW	TH
NM	FI	FO	FO	TH	TH	TW	TW	TH	TH
NS	FO	FO	TH	TH	TW	TW	TH	TH	FO
ZE	FO	TH	TH	TW	FO	TH	TH	FO	FO
PS	TH	TH	TW	TW	TH	TH	FO	FO	FI
PM	TH	TW	TW	TH	TH	FO	FO	FI	FI
PR	TW	TW	TH	TH	FO	FO	FI	FI	FI
PL	TW	TH	TH	FO	FO	FI	FI	FI	FI

**Table:** (b) Δ*c*(*t*) and Δ*m*(*t*)

Δ*f*(*t*)	Δ*f*(*t* − 1)								
	NL	NR	NM	NS	ZE	PS	PM	PR	PL
NL	NL	NR	NR	NM	NM	NS	NS	ZE	ZE
NR	NR	NR	NM	NM	NS	NS	ZE	ZE	PS
NM	NR	NM	NM	NS	NS	ZE	ZE	PS	PS
NS	NM	NM	NS	NS	ZE	ZE	PS	PS	PM
ZE	NM	NS	NS	ZE	ZE	PS	PS	PM	PM
PS	NS	NS	ZE	ZE	PS	PS	PM	PM	PR
PM	NS	ZE	ZE	PS	PS	PM	PM	PR	PR
PR	ZE	ZE	PS	PS	PM	PM	PR	PR	PL
PL	ZE	PS	PS	PM	PM	PR	PR	PL	PL

**Table:** (a) Δtc(*t*)

*Z*(*X*, *Y*)		*X*								
		−4	−3	−2	−1	0	1	2	3	4
*Y*	−4	5	5	5	4	4	3	3	2	2
	−3	5	5	4	4	3	3	2	2	3
	−2	5	4	4	3	3	2	2	3	3
	−1	4	4	3	3	2	2	3	3	4
	0	4	3	3	2	4	3	3	4	4
	1	3	3	2	2	3	3	4	4	5
	2	3	2	2	3	3	4	4	5	5
	3	2	2	3	3	4	4	5	5	5
	4	2	3	3	4	4	5	5	5	5

**Table:** (b) Δ*c*(*t*) and Δ*m*(*t*)

*Z*(*X*, *Y*)		*X*								
		−4	−3	−2	−1	0	1	2	3	4
*Y*	−4	−4	−3	−3	−2	−2	−1	−1	−0	+0
	−3	−3	−3	−2	−2	−1	−1	−0	+0	1
	−2	−3	−2	−2	−1	−1	−0	+0	1	1
	−1	−2	−2	−1	−1	−0	+0	1	1	2
	0	−2	−1	−1	−0	+0	1	1	2	2
	1	−1	−1	−0	+0	1	1	2	2	3
	2	−1	−0	+0	1	1	2	2	3	3
	3	−0	+0	1	1	2	2	3	3	4
	4	+0	1	1	2	2	3	3	4	4

**Table 7:** Input data for numerical example.

Number of jobs	Number of products	Number of parts	Number of lathe machines, CNC, and robots	Number of machine tools
50	4	20	5	5

**Table 8:** Fuzzy variables and initialization of parameters.

*X*_*i*_	*J*_*i*_	*P*.NO	A.S	S.No	*P*(*X*_*i*_)	*M*(*X*_*i*_)	OP			T.t		
1	*J*_ 1_	*P*.NO_1_	AS_0_	A	*P*_ 0_	*M*_ 0_	1.50	2	2.50	0.50	1	1.5
2	*J*_ 2_	*P*.NO_1_	AS_0_	A	*P*_ 0_	*M*_ 1_	2.50	3	3.50	4.50	5	5.5
3	*J*_ 3_	*P*.NO_1_	AS_0_	A	*P*_ 0_	*M*_ 2_	0.50	1	1.50	0.50	1	1.5
4	*J*_ 4_	*P*.NO_1_	AS_1_	B	*P*_ 1_	*M*_ 0_	0.50	1	1.50	1.50	2	2.5
5	*J*_ 5_	*P*.NO_1_	AS_1_	B	*P*_ 1_	*M*_ 2_	1.50	2	2.50	2.50	3	3.5
6	*J*_ 6_	*P*.NO_1_	AS_1_	A	*P*_ 2_	*M*_ 0_	1.50	2	2.50	2.50	3	3.5
7	*J*_ 7_	*P*.NO_1_	AS_1_	A	*P*_ 2_	*M*_ 2_	0.50	1	1.50	2.50	3	3.5
8	*J*_ 8_	*P*.NO_1_	AS_2_	C	*P*_ 3_	*M*_ 3_	1.50	2	2.50	3.50	4	4.5
9	*J*_ 9_	*P*.NO_1_	AS_2_	A	*P*_ 3_	*M*_ 1_	2.50	3	3.50	4.50	5	5.5
10	*J*_ 10_	*P*.NO_1_	AS_2_	A	*P*_ 3_	*M*_ 4_	1.50	2	2.50	0.50	1	1.5
11	*J*_ 11_	*P*.NO_2_	AS_0_	E	*P*_ 4_	*M*_ 2_	3.50	4	4.50	1.50	2	2.5
12	*J*_ 12_	*P*.NO_2_	AS_0_	C	*P*_ 4_	*M*_ 1_	2.50	3	3.50	0.50	1	1.5
13	*J*_ 13_	*P*.NO_2_	AS_0_	E	*P*_ 4_	*M*_ 0_	1.50	2	2.50	2.50	3	3.5
14	*J*_ 14_	*P*.NO_2_	AS_0_	E	*P*_ 5_	*M*_ 1_	0.50	1	1.50	1.50	2	2.5
15	*J*_ 15_	*P*.NO_2_	AS_0_	B	*P*_ 5_	*M*_ 2_	0.50	1	1.50	0.50	1	1.5
16	*J*_ 16_	*P*.NO_2_	AS_0_	B	*P*_ 6_	*M*_ 0_	1.50	2	2.50	3.50	4	4.5
17	*J*_ 17_	*P*.NO_2_	AS_0_	B	*P*_ 6_	*M*_ 3_	1.50	2	2.50	0.50	1	1.5
18	*J*_ 18_	*P*.NO_2_	AS_0_	B	*P*_ 6_	*M*_ 1_	2.50	3	3.50	1.50	2	2.5
19	*J*_ 19_	*P*.NO_2_	AS_1_	C	*P*_ 7_	*M*_ 0_	2.50	3	3.50	2.50	3	3.5
20	*J*_ 20_	*P*.NO_2_	AS_1_	C	*P*_ 7_	*M*_ 1_	0.50	1	1.50	1.50	2	2.5
21	*J*_ 21_	*P*.NO_2_	AS_2_	A	*P*_ 8_	*M*_ 3_	0.50	1	1.50	0.50	1	1.5
22	*J*_ 22_	*P*.NO_2_	AS_2_	A	*P*_ 8_	*M*_ 2_	4.50	5	5.50	1.50	2	2.5
23	*J*_ 23_	*P*.NO_2_	AS_3_	D	*P*_ 9_	*M*_ 3_	2.50	3	3.50	2.50	3	3.5
24	*J*_ 24_	*P*.NO_2_	AS_3_	B	*P*_ 9_	*M*_ 2_	1.50	2	2.50	1.50	2	2.5
25	*J*_ 25_	*P*.NO_2_	AS_3_	D	*P*_ 9_	*M*_ 4_	0.50	1	1.50	4.50	5	5.5
26	*J*_ 26_	*P*.NO_3_	AS_0_	B	*P*_ 10_	*M*_ 1_	3.50	4	4.50	0.50	1	1.5
27	*J*_ 27_	*P*.NO_3_	AS_0_	A	*P*_ 10_	*M*_ 0_	2.50	3	3.50	0.50	1	1.5
28	*J*_ 28_	*P*.NO_3_	AS_0_	C	*P*_ 10_	*M*_ 2_	1.50	2	2.50	2.50	3	3.5
29	*J*_ 29_	*P*.NO_3_	AS_1_	A	*P*_ 11_	*M*_ 4_	0.50	1	1.50	1.50	2	2.5
30	*J*_ 30_	*P*.NO_3_	AS_1_	A	*P*_ 11_	*M*_ 1_	1.50	2	2.50	1.50	2	2.5
31	*J*_ 31_	*P*.NO_3_	AS_1_	C	*P*_ 12_	*M*_ 0_	0.50	1	1.50	1.50	2	2.5
32	*J*_ 32_	*P*.NO_3_	AS_1_	C	*P*_ 12_	*M*_ 1_	1.50	2	2.50	0.50	1	1.5
33	*J*_ 33_	*P*.NO_3_	AS_1_	C	*P*_ 12_	*M*_ 2_	2.50	3	3.50	2.50	3	3.5
34	*J*_ 34_	*P*.NO_3_	AS_2_	B	*P*_ 13_	*M*_ 0_	3.50	4	4.50	3.50	4	4.5
35	*J*_ 35_	*P*.NO_3_	AS_2_	E	*P*_ 13_	*M*_ 2_	4.50	5	5.50	4.50	5	5.5
36	*J*_ 36_	*P*.NO_3_	AS_2_	E	*P*_ 13_	*M*_ 1_	1.50	2	2.50	0.50	1	1.5
37	*J*_ 37_	*P*.NO_3_	AS_3_	E	*P*_ 14_	*M*_ 3_	0.50	1	1.50	4.50	5	5.5
38	*J*_ 38_	*P*.NO_3_	AS_3_	B	*P*_ 14_	*M*_ 4_	2.50	3	3.50	2.50	3	3.5
39	*J*_ 39_	*P*.NO_3_	AS_4_	B	*P*_ 15_	*M*_ 1_	4.50	5	5.50	1.50	2	2.5
40	*J*_ 40_	*P*.NO_3_	AS_4_	B	*P*_ 15_	*M*_ 3_	3.50	4	4.50	0.50	1	1.5
41	*J*_ 41_	*P*.NO_4_	AS_0_	A	*P*_ 16_	*M*_ 2_	3.50	4	4.50	0.50	1	1.5
42	*J*_ 42_	*P*.NO_4_	AS_0_	D	*P*_ 16_	*M*_ 0_	2.50	3	3.50	2.50	3	3.5
43	*J*_ 43_	*P*.NO_4_	AS_0_	A	*P*_ 16_	*M*_ 1_	1.50	2	2.50	1.50	2	2.5
44	*J*_ 44_	*P*.NO_4_	AS_0_	B	*P*_ 17_	*M*_ 0_	0.50	1	1.50	3.50	4	4.5
45	*J*_ 45_	*P*.NO_4_	AS_0_	B	*P*_ 17_	*M*_ 3_	0.50	1	1.50	0.50	1	1.5
46	*J*_ 46_	*P*.NO_4_	AS_1_	A	*P*_ 18_	*M*_ 2_	3.50	4	4.50	1.50	2	2.5
47	*J*_ 47_	*P*.NO_4_	AS_1_	B	*P*_ 18_	*M*_ 1_	2.50	3	3.50	1.50	2	2.5
48	*J*_ 48_	*P*.NO_4_	AS_2_	B	*P*_ 19_	*M*_ 0_	1.50	2	2.50	2.50	3	3.5
49	*J*_ 49_	*P*.NO_4_	AS_2_	A	*P*_ 19_	*M*_ 4_	0.50	1	1.50	1.50	2	2.5
50	*J*_ 50_	*P*.NO_4_	AS_2_	B	*P*_ 19_	*M*_ 3_	3.50	4	4.50	2.50	3	3.5

**Table 9:** Summary of the final results based on existing and optimized data.

	Existing data	Optimized data
	(Opt., Med., Pes.)	(Opt., Med., Pes.)
Total scheduling time	(166, 250, 266)	(62.5, 73, 88.5)
Total setup number (No.)	(44, 44, 44)	(37, 37, 39)
Total efficient frontiers	(105, 147, 155)	(49.25, 54.5, 63.75)
Total scheduling time with setup time	(254, 338, 354)	(135, 145, 168)
Total operation setup time	(88, 88, 88)	(72, 72, 78)
Total changing setup cost ($)	(3520, 3520, 3520)	(2880, 2880, 3120)
Total units produced per day	(2.89, 1.92, 1.80)	(7.62, 6.58, 5.33)
Total efficiency (%)	(11.45%, 10.32%, 10.90%)	(30.16%, 35.34%, 32.22%)
Total idle time (%)	(88.55%, 89.68%, 89.10%)	(69.84%, 64.66%, 67.78%)

**Table 10:** Comparison of optimum fitness values with respect to the number of generations and time taken to achieve convergence for various tournament candidates and crossover and mutation rates.

	Tournament candidates						
	Tournament candidates (2)		Tournament candidates (3)		Tournament candidates (4)	Tournament candidates (5)	Tournament candidates (*fuzzy)
Value	57		57		56.5	56.5	56
Generation	145		96		272	73	60
Time	1.48		1.48		1.46	1.46	1.45

	Crossover rate						
	Crossover rate (0.25)	Crossover rate (0.5)	Crossover rate (0.7)	Crossover rate (0.8)	Crossover rate (0.9)	Crossover rate (1)	Crossover rate (*fuzzy)

Value	56	58.5	56.5	56.5	56.5	56.5	55
Generation	269	45	392	127	80	64	79
Time	6.99	1.17	10.19	3.30	2.08	1.66	2.50

	Mutation rate						
	Mutation rate (0.0)	Mutation rate (0.01)	Mutation rate (0.02)	Mutation rate (0.03)	Mutation rate (0.04)	Mutation rate (0.05)	Mutation rate (*fuzzy)

Value	61	57.5	56.5	56	55.5	55.5	55
Generation	48	188	154	527	529	479	147
Time	1.25	4.88	4.01	13.70	13.75	12.45	3.82

**Table 11:** Comparison of GA versus FGA with respect to fitness value, number of generations, and time taken to achieve convergence for various control parameters.

	GA	FGA
Chromosome size	50	FES
Population size	100	FES
Tournament candidate	3	ALDFC
Crossover	0.5	ALDFC
Mutation	0.02	ALDFC
Generation	243	83
Value	57.5	54.5
Time	6.31′′	2.15′′
